# Cortisol–CX_3_CL1 association and altered cytokine–chemokine profiles in emergency medical services personnel

**DOI:** 10.3389/fimmu.2026.1903713

**Published:** 2026-07-10

**Authors:** Elena R. Serrano-Ibáñez, María Flores-López, Laura Martín-Chaves, Tania Corrás-Vázquez, Inés Antúnez-Muñoz, Javier Samper-Zapata, Ada del Mar Carmona-Segovia, Raquel Reviriego, Manuel Jiménez-Navarro, Fernando Rodríguez de Fonseca, Antonia Serrano, Francisco Javier Pavón-Morón

**Affiliations:** 1 IBIMA Plataforma BIONAND, Instituto de Investigación Biomédica de Málaga, Málaga, Spain; 2 Facultad de Psicología y Logopedia, Universidad de Málaga, Málaga, Spain; 3 Área de Psicología, Universidad Isabel I, Burgos, Spain; 4 Unidad de Gestión Clínica de Salud Mental, Hospital Regional Universitario de Málaga, Málaga, Spain; 5 Área del Corazón, Hospital Universitario Virgen de la Victoria, Málaga, Spain; 6 Facultad de Medicina, Universidad de Málaga, Málaga, Spain; 7 Facultad de Ciencia, Tecnología y Gestión de la Salud, Universidad Intercontinental de la Empresa, Santiago de Compostela, Spain; 8 CIBERCV, CIBER de Enfermedades Cardiovasculares, Instituto de Salud Carlos III, Madrid, Spain; 9 Unidad Clínica de Neurología, Hospital Regional Universitario de Málaga, Málaga, Spain

**Keywords:** chemokines, cortisol, CX_3_CL1, cytokines, emergency medical services, fractalkine, inflammation, psychological distress

## Abstract

**Background:**

Psychological distress may be associated with systemic immune regulation through neuroendocrine–immune pathways. Emergency medical services (EMS) personnel represent a high-demand occupational group, but their circulating cytokine and chemokine profiles and their relationship with psychological distress remain poorly characterized. This exploratory cross-sectional study examined inflammatory and physiological markers in EMS personnel compared with matched controls, with particular attention to the association between cortisol and CX_3_CL1 and exploratory sex-related patterns.

**Methods:**

Seventy-eight participants, including 39 EMS personnel and 39 matched controls, underwent assessment of blood pressure, plasma cortisol, cytokines, chemokines, soluble adhesion molecules, and an exploratory panel of cardiovascular-related proteins. Psychological distress, including depression, anxiety, and stress symptoms, was assessed using the Depression, Anxiety and Stress Scale-21 in EMS personnel. Plasma concentrations of inflammatory mediators were analyzed after log10 transformation using two-way analysis of variance with group and sex as fixed factors. Spearman correlation analyses were used to explore associations between cortisol and inflammatory mediators.

**Results:**

The EMS group showed higher systolic blood pressure and cortisol levels than the control group. The EMS group also exhibited a broad cytokine–chemokine alteration characterized by increased concentrations of several cytokines, including GM-CSF, IFN-α, IL-1α, IL-1β, IL-6, IL-10, and IL-13, and chemokines, including CCL2 and CX_3_CL1/fractalkine. In contrast, circulating E-selectin, P-selectin, and sICAM-1 were reduced in the EMS group. Most cytokines and chemokines formed a highly intercorrelated inflammatory cluster. However, CX_3_CL1 showed a more independent correlation pattern and was selectively associated with cortisol, whereas cortisol was not associated with the broader cytokine–chemokine network. This cortisol–CX_3_CL1 association was more evident among women. Within the EMS group, women reported higher anxiety and stress scores than men, whereas men showed higher systolic and diastolic blood pressure.

**Conclusion:**

EMS personnel exhibited an altered systemic cytokine–chemokine profile together with physiological changes and exploratory sex-related patterns. The selective association between cortisol and CX_3_CL1 suggests that circulating CX_3_CL1/fractalkine may reflect a stress-sensitive chemokine and a candidate marker of neuroendocrine–immune interaction. These findings support the relevance of integrating psychological, endocrine, and immune markers to characterize biological responses to psychological distress in high-demand healthcare populations.

## 1 Introduction

Psychological distress is a prevalent and growing concern worldwide, particularly in occupations characterized by high demands, intense emotional pressure, and critical decision-making (1). Emergency medical services (EMS) personnel represent a high-demand healthcare population frequently exposed to acute and unpredictable stressors, including irregular work schedules, life-threatening situations, rapid clinical decision-making, and high emotional labor. These conditions may contribute to psychological distress, encompassing symptoms of stress, anxiety, and depression. Psychological distress in healthcare workers has been associated not only with mental health outcomes, such as anxiety, burnout, and post-traumatic stress disorder, but also with physiological alterations, particularly in immune and inflammatory processes (2–4). Critical situations such as the COVID-19 pandemic have further intensified these demands, increasing the psychological burden among healthcare workers and highlighting the need to better understand the biological pathways that may underlie stress-related health vulnerability in this essential workforce (5–7).

Psychological distress may be associated with health outcomes through neuroendocrine–immune pathways, particularly by activating the hypothalamic–pituitary–adrenal (HPA) axis. Activation of this system induces physiological responses aimed at restoring homeostasis, but sustained or dysregulated activation may contribute to immune alterations and changes in circulating inflammatory mediators (8, 9). Cytokines are key signaling molecules that coordinate immune responses, whereas chemokines regulate immune cell recruitment, migration, and tissue surveillance (10, 11). Although these mediators are essential for host defense and tissue repair, persistent alterations in cytokine and chemokine profiles have been linked to cardiovascular disease, metabolic syndrome, and neuropsychiatric disorders (12–14). In addition, cell adhesion molecules mediate leukocyte adhesion and transmigration across the vascular endothelium and contribute to inflammatory and vascular homeostasis (15–17). Their soluble forms may therefore provide complementary information on endothelial and immune regulation, processes that are particularly relevant for cardiovascular vulnerability in populations exposed to sustained psychological demands (18, 19).

These biological processes are especially relevant in healthcare workers, a population exposed to demanding and emotionally intense occupational contexts. Previous studies have shown that individuals in high-demand work settings may exhibit altered circulating inflammatory mediators, including cytokines and chemokines, which may serve as biological correlates of stress exposure and potential indicators of future health risk (20–23). However, evidence specifically characterizing cytokine, chemokine, and adhesion molecule profiles in EMS personnel remains limited, as most available studies have focused on broader healthcare populations or other occupational groups (24, 25). This gap limits our understanding of whether EMS personnel show a distinct cytokine–chemokine profile and whether specific immune mediators are associated with endocrine markers of stress, such as cortisol.

Sex-related differences in neuroendocrine and immune responses may further influence the biological correlates of psychological distress. Evidence suggests that men and women may differ in stress perception, HPA axis regulation, immune responses, and vulnerability to stress-related health outcomes (26). However, whether sex-related patterns are present in EMS personnel, and whether they involve specific associations between psychological distress, cortisol, and inflammatory mediators, remains insufficiently explored. This is particularly relevant when evaluating chemokines such as CX_3_CL1/fractalkine, which may behave differently from broader cytokine networks and could provide insight into selective neuroendocrine–immune associations. CX_3_CL1/fractalkine is of particular interest because, unlike most chemokines, it exists in both membrane-bound and soluble forms, signals through a single receptor (CX_3_CR1) (27), and has been implicated in microglial regulation and the neuroendocrine response to stress (28, 29), which may link it more closely to HPA-axis activity than the broader cytokine network.

The primary aim of this exploratory study was to examine immune and physiological markers in EMS personnel compared with matched controls, and to assess their associations with psychological distress. Specifically, we evaluated plasma concentrations of cytokines, chemokines, soluble adhesion molecules, and cortisol, together with an exploratory panel of cardiovascular-related proteins. Given the potential relevance of chemokine-mediated immune regulation, particular attention was paid to CX_3_CL1/fractalkine and its association with cortisol. In addition, we explored whether these biological patterns differed by sex. By characterizing these associations, this study aims to contribute to a better understanding of psychological, endocrine, and immune correlates of distress in a high-demand healthcare population.

## 2 Materials and methods

### 2.1 Study design, participants, and ethical considerations

This exploratory cross-sectional study included 78 participants divided into two groups: 39 EMS personnel (EMS group) and 39 control subjects (control group). Participants in the EMS group were recruited from the *Centro de Emergencias Sanitarias 061 en Málaga* (Public EMS Center 061 of Malaga, Spain). Control subjects were selected from volunteers who donated data and plasma samples to the *Red de Biobancos del Instituto de Salud Carlos III* (Valdecilla Biobank, Santander, Spain). Controls were matched with the EMS group by age, body mass index (BMI), and sex.

Eligible participants were men and women aged ≥ 18 years. Inclusion criteria for the EMS group required active employment in emergency medical services. Exclusion criteria for all participants included infectious diseases (i.e., COVID-19, HIV, hepatitis B, and hepatitis C), recent vaccination (within the previous 3 months), pregnancy or breastfeeding, and cognitive or severe language limitations that could interfere with study participation.

Participation was voluntary, and all participants provided written informed consent before inclusion. The study and recruitment procedures were approved by the Ethics Committee of the *Portal de Ética de la Investigación Biomédica de Andalucía* (*PEIBA; Consejería de Salud y Familias, Junta de Andalucía*).

### 2.2 Biological, clinical, and psychological assessment

Age, sex, and BMI were collected as demographic, biological, and anthropometric variables. In the EMS group, additional clinical and lifestyle information was recorded, including tobacco use, alcohol consumption, current medical conditions or comorbidities, and the use of psychotropic or other prescribed medications, including anti-inflammatory drugs. Information on recent acute infection other than the viral infections specifically screened for was not systematically collected; however, when available, it was considered in the interpretation of inflammatory marker findings.

Blood pressure was measured in millimeters of mercury (mmHg) using a sphygmomanometer under resting conditions, and both systolic and diastolic blood pressure values were recorded. Measurements were obtained after 5 minutes of seated rest, with the participant’s back supported, feet flat on the floor, legs uncrossed, and the arm supported at heart level. An appropriately sized upper-arm cuff, selected according to mid-arm circumference, was used. Three consecutive readings were obtained at 1-minute intervals, and the mean of the final two readings was used for analysis.

Psychological distress was assessed using the short-form version of the Depression, Anxiety, and Stress Scale (DASS-21), adapted and validated in Spanish (30). This self-report questionnaire consists of 21 items grouped into three dimensions: depression, anxiety, and stress. Symptom severity is evaluated using a four-point Likert scale ranging from 0 (never) to 3 (most of the time). Participants responded according to how frequently they had experienced each state during the previous week. Internal consistency in the present sample was high, with Cronbach’s α = 0.83 for depression, 0.85 for anxiety, and 0.88 for stress.

### 2.3 Blood collection and plasma processing

After overnight fasting, venous blood samples were obtained in the morning by experienced nurses. Blood samples were collected into 9-mL K_2_ EDTA tubes (BD, Franklin Lakes, NJ, USA) and immediately centrifuged at 2200 × g for 10 minutes at 4 °C to obtain plasma. Each plasma sample was tested for infectious diseases using rapid tests for HIV, hepatitis B, hepatitis C (Strasbourg, Cedex, France), and SARS-CoV-2 (Bio-Connect, Huissen, The Netherlands). No positive results were detected in the study sample. Plasma aliquots were stored at -80°C until further analysis. In EMS personnel, blood was drawn at the start of the daytime shift, within a defined morning window (08:00–10:00 h). According to the biobank records, control samples were likewise collected in the morning, within a comparable time window (07:00–11:00 h), supporting the comparability of cortisol measurements between groups.

None of the EMS participants had worked a night shift immediately before sampling, which minimizes the influence of recent night duty on cortisol levels.

### 2.4 Immunoassays

All plasma immunoassays were performed in duplicate across two 96-well plates per assay, according to the manufacturers’ instructions. Control and EMS samples were alternated and approximately evenly distributed across the plates, with the order of individual samples within each group randomized, to minimize potential plate-related variability. Laboratory personnel were blinded to group allocation during the assays. Each plate included its corresponding standard curve, and analyte concentrations were calculated from the curve generated on the same plate.

#### 2.4.1 ELISA for cortisol

Plasma cortisol concentrations were quantified using a high-sensitivity enzyme-linked immunosorbent assay (ELISA) kit (#HEA462Ge, Cloud-Clone Corp., Katy, TX, USA). The assay was performed using 50 µL of plasma per well. Absorbance readings were obtained at 450 nm, and cortisol concentrations were calculated from the standard curve in ng/mL. Values were converted to nmol/L for reporting using the following conversion factor: nmol/L = ng/mL × 2.759. The inter-assay and intra-assay coefficients of variation (CVs) were 6.1% and 9.0%, respectively.

#### 2.4.2 Multiplexed bead-based immunoassays for inflammatory mediators

Plasma concentrations of cytokines, chemokines, and soluble adhesion molecules were quantified using ProcartaPlex™ Human Inflammation Panels and ProcartaPlex™ Simplex Kits (#EPX200-12185–901 and #EPX01A-12121-901, Thermo Fisher Scientific, Waltham, MA, USA).

The cytokine panel included granulocyte-macrophage colony-stimulating factor (GM-CSF), interferon alpha (IFN-α), IFN gamma (IFN-γ), interleukin 1 alpha (IL-1α), IL-1 beta (IL-1β), IL-4, IL-6, IL-10, IL-12p70, IL-13, IL-17A, and tumor necrosis factor alpha (TNF-α). The chemokine panel included C-X-C motif chemokine ligand 8 (CXCL8, IL-8), CXCL10 (IFN-γ-induced protein 10, IP-10), C-C motif chemokine ligand 2 (CCL2, monocyte chemoattractant protein-1, MCP-1), CCL3 (macrophage inflammatory protein-1 alpha, MIP-1α), CCL4 (MIP-1β), and C-X3-C motif ligand 1 (CX_3_CL1, fractalkine). The soluble adhesion and inflammatory response-related proteins included soluble intercellular adhesion molecule-1 (sICAM-1), CD62E (E-selectin), and CD62P (P-selectin).

Measurements were performed using 50 µL of plasma per well and a Luminex 200 instrument system (Thermo Fisher Scientific, Waltham, MA, USA). Median fluorescence intensity values were acquired using the Luminex platform, and analyte concentrations were expressed in pg/mL or ng/mL, as appropriate. For all analytes, inter-assay CVs ranged from 6.5% to 10.1%, and intra-assay CVs ranged from 5.0% to 9.2%. Four samples, three from the control group and one from the EMS group, were excluded because of technical issues.

#### 2.4.3 Multiplexed bead-based immunoassays for cardiovascular-related proteins

As an exploratory extension of the inflammatory profile, plasma concentrations of five cardiovascular-related proteins were additionally quantified: α2-macroglobulin (A2M), C-reactive protein (CRP), serum amyloid P component (SAP), haptoglobin (HPTGN), and α1-acid glycoprotein (AGP). These proteins were measured using the MILLIPLEX MAP Human Cardiovascular Disease (CVD) Magnetic Bead Panel 3 (#HCVD3MAG-67K, Merck KGaA, Darmstadt, Germany).

All assays were performed with 25 μL of plasma per well in a Luminex 200 system (Thermo Fisher Scientific, Waltham, MA, USA). Analyte concentrations were expressed in μg/mL or ng/mL according to kit specifications. Intra-assay CVs were 8.47% for A2M, 12.44% for CRP, 14.65% for SAP, 12.51% for HPTGN, and 9.19% for AGP.

### 2.5 Statistical analysis

Data are presented as number and percentage of subjects [n (%)], mean and standard deviation (mean ± SD), or median and interquartile range [median (IQR, 25th - 75th percentile)], as appropriate. Distributional assumptions were assessed before group comparisons.

Categorical variables were analyzed using Fisher’s exact test. Continuous variables were compared between two independent groups using Student’s *t* test for normally distributed variables and the Mann–Whitney *U* test for non-normally distributed variables. For the significant between-group differences, effect sizes were estimated as Cohen’s *d* with 95% confidence intervals (95% CIs) and are reported in the main text; Cohen’s *d* was computed on raw values for cortisol and on log_10_-transformed values for the inflammatory mediators, consistent with their distributional treatment.

For factorial analyses, concentrations of cortisol, cytokines, chemokines, and soluble adhesion molecules were log_10_-transformed to reduce skewness and improve model assumptions, and evaluated using two-way analysis of variance (ANOVA) with group (EMS vs. control; factor 1 = f1) and sex (men vs. women; factor 2 = f2) as fixed factors. Main effects of group and sex, and group × sex interactions, were examined; when a significant interaction was detected, *post hoc* comparisons were performed.

Associations between cortisol and inflammatory mediators, and the internal structure of the mediator network, were explored using Spearman’s rank correlation coefficient (*r_s_*).

Given the broad biomarker panel and the exploratory nature of the study, nominal *p*-values are reported throughout the manuscript and interpretation was based primarily on coherent biomarker patterns rather than isolated significant findings. As a sensitivity analysis, Benjamini–Hochberg false discovery rate (FDR)–adjusted *p*-values were computed within each predefined analyte family and are reported in **Supplementary Table 1** and **Supplementary Results S1**, with the findings that remained significant after correction indicated. Primary outcomes were the between-group differences in cytokines, chemokines, and soluble adhesion molecules and the association between cortisol and CX_3_CL1; sex-related patterns and the cardiovascular-related protein panel were predefined as secondary, exploratory outcomes.

Test statistic values were reported where applicable. A two-sided *p* < 0.05 was considered statistically significant. Statistical analyses were performed using GraphPad Prism version 5.04 (GraphPad Software, San Diego, CA, USA) and IBM SPSS Statistical version 23 (IBM, Armonk, NY, USA).

## 3 Results

### 3.1 Biological and physiological characteristics

**Table 1** presents the baseline biological and physiological characteristics of the sample. The median age of participants in the EMS group was 48 years, 69% were women, and the median BMI was 24.5 kg/m^2^. No statistically significant differences were found between groups in sex, age, or BMI.

**Table 1:** Biological characteristics of the sample.

Variable		Group		*p*-value
		Control	EMS	
Participants (n)		39	39	>0.999 ^a^
Sex [n (%)]	Men	16 (41.0)	12 (30.8)	0.479 ^a^
	Women	23 (59.0)	27 (69.2)	
Age (years) [median (IQR)]		47 (44 - 51)	48 (44-56)	0.278 ^b^
BMI (kg/m^2^) [median (IQR)]		24.6 (23.0-27.7)	24.5 (22.9-27.8)	0.815 ^b^
Blood pressure (mmHg) [median (IQR)]	Systolic	120 (107-122)	134 (122-146)	**<0.001** ^b^
	Diastolic	80 (61-85)	80 (76-94)	>0.999 ^b^
Cortisol (nmol/L) (mean ± SD)		605.58 ± 166.42	763.54 ± 166.03	**<0.001** ^c^

**^a^**Data were analyzed using the Fisher’s exact test.

**^b^**Data were analyzed using the Man–Whitney *U* test.

**^c^**Data were analyzed using the Student’s *t* test.

BMI, body mass index; EMS, emergency medical services; IQR, interquartile range; SD, standard deviation.

Bold values indicate statistical significance.

Significant group differences were observed in systolic blood pressure and plasma cortisol levels. The EMS group showed higher systolic blood pressure than the control group (134 vs. 120 mmHg, *p* < 0.001; approximate Cohen’s *d* ≈ 0.91, IC95% 0.45–1.38), whereas no significant difference was detected in diastolic blood pressure. Plasma cortisol levels were also significantly higher in the EMS group than in the control group (763.54 ± 166.03 vs. 605.58 ± 166.42 nmol/L, *p* < 0.001; Cohen’s *d* = 0.95, 95%CI 0.48–1.42).

### 3.2 Plasma inflammatory mediator profile

Plasma concentrations of cytokines, chemokines, and soluble adhesion molecules were quantified in the full sample and compared between groups. In the corresponding figures, bars show the mean ± SD (normally distributed analytes) or the median with IQR (non-normally distributed analytes), with individual data points overlaid on the raw scale; full summary statistics are presented in **Supplementary Table 1**.

#### 3.2.1 Cytokines

**Figure 1:**
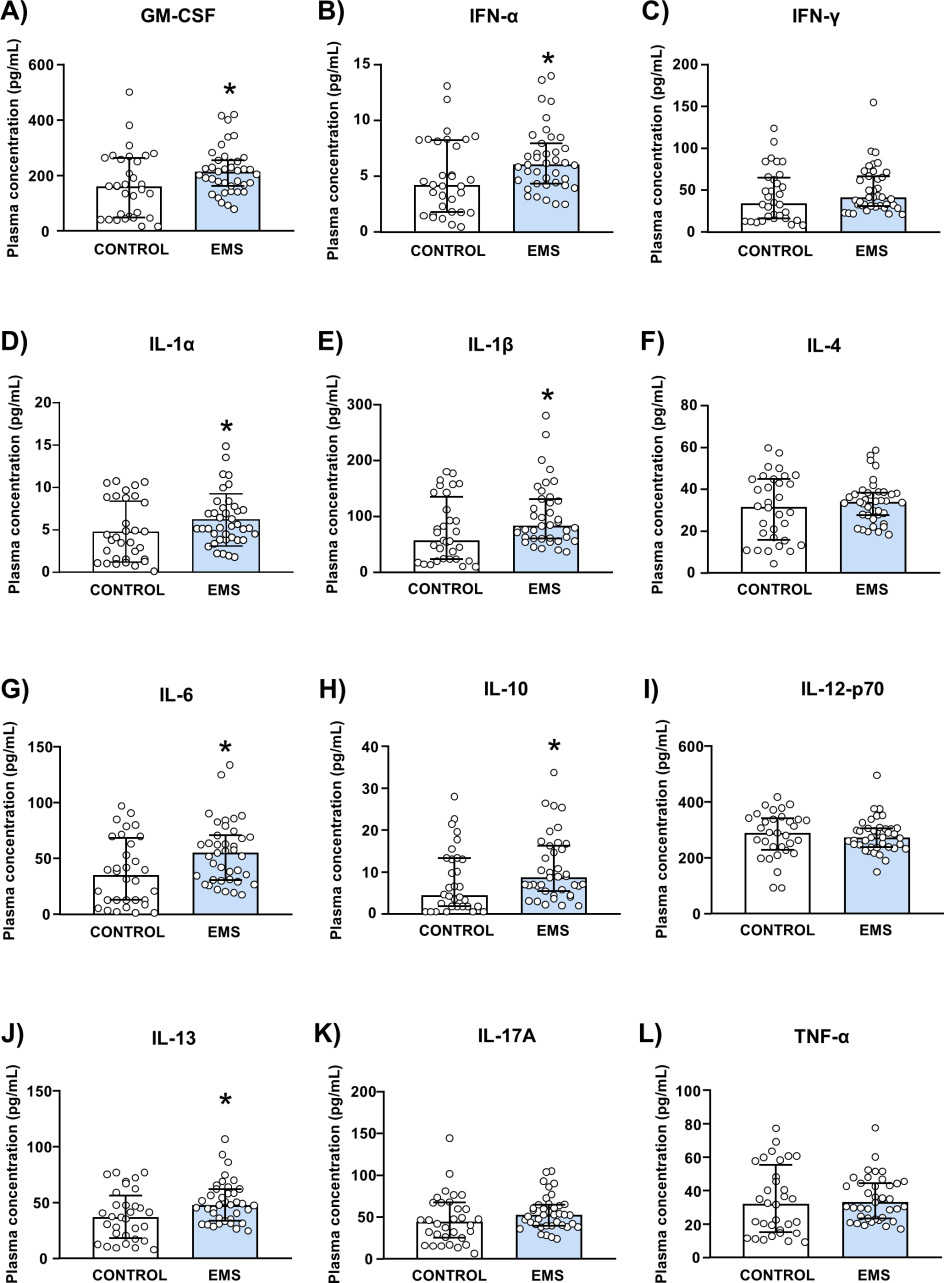
Plasma cytokine concentrations in controls and EMS personnel. **(A)** GM-CSF (pg/mL). **(B)** IFN-α (pg/mL). **(C)** IFN-γ (pg/mL). **(D)** IL-1α (pg/mL). **(E)** IL-1β (pg/mL). **(F)** IL-4 (pg/mL). **(G)** IL-6 (pg/mL). **(H)** IL-10 (pg/mL). **(I)** IL-12-p70 (pg/mL). **(J)** IL-13 (pg/mL). **(K)** IL-17A (pg/mL). **(L)** TNF-α (pg/mL). Concentrations are shown on the raw scale. Bars represent the mean with SD for normally distributed variables (GM-CSF, IL-4, and IL-13) and the median with IQR for non-normally distributed variables (IFN-α, IFN-γ, IL-1α, IL-1β, IL-6, IL-10, IL-12-p70, IL-17A, and TNF-α). Data were analyzed using Student’s *t* test or the Mann-Whitney *U* test according to distribution. (*) denotes nominal *p* < 0.05 compared with the control group. Numerical values and statistical results are reported in **Supplementary Table 1**.

The EMS group exhibited higher circulating concentrations of several cytokines compared with the control group. Significant group differences were observed for GM-CSF (*t* = 2.176, *p* = 0.033; Cohen’s *d* = 0.77, 95%CI 0.29–1.26; **Figure 1A**), IFN-α (*U* = 423, *p* = 0.020; Cohen’s *d* = 0.73, 95%CI 0.25–1.21; **Figure 1B**), IL-1α (*U* = 450.5, *p* = 0.045; Cohen’s *d* = 0.64, 95%CI 0.16–1.12; **Figure 1D**), IL-1β (*U* = 431.5, *p* = 0.026; Cohen’s *d* = 0.73, 95%CI 0.24–1.21; **Figure 1E**), IL-6 (*U* = 420.5, *p* = 0.018; Cohen’s *d* = 0.84, 95%CI 0.35–1.33; **Figure 1G**), IL-10 (*U* = 424, *p* = 0.020; Cohen’s *d* = 0.70, 95%CI 0.22–1.18; **Figure 1H**), and IL-13 (*t* = 2.409, *p* = 0.019; Cohen’s *d* = 0.75, 95%CI 0.27–1.24; **Figure 1J**). All these cytokines were elevated in the EMS group compared with the control group. After FDR correction within the cytokine family, none of these differences remained significant (**Supplementary Table 1**), consistent with their interpretation as a coherent pattern rather than isolated effects.

No significant between-group differences were observed for IFN-γ (**Figure 1C**), IL-4 (**Figure 1F**), IL-12-p70 (**Figure 1I**), IL-17A (**Figure 1K**), and TNF-α (**Figure 1L**).

#### 3.2.2 Chemokines

**Figure 2:**
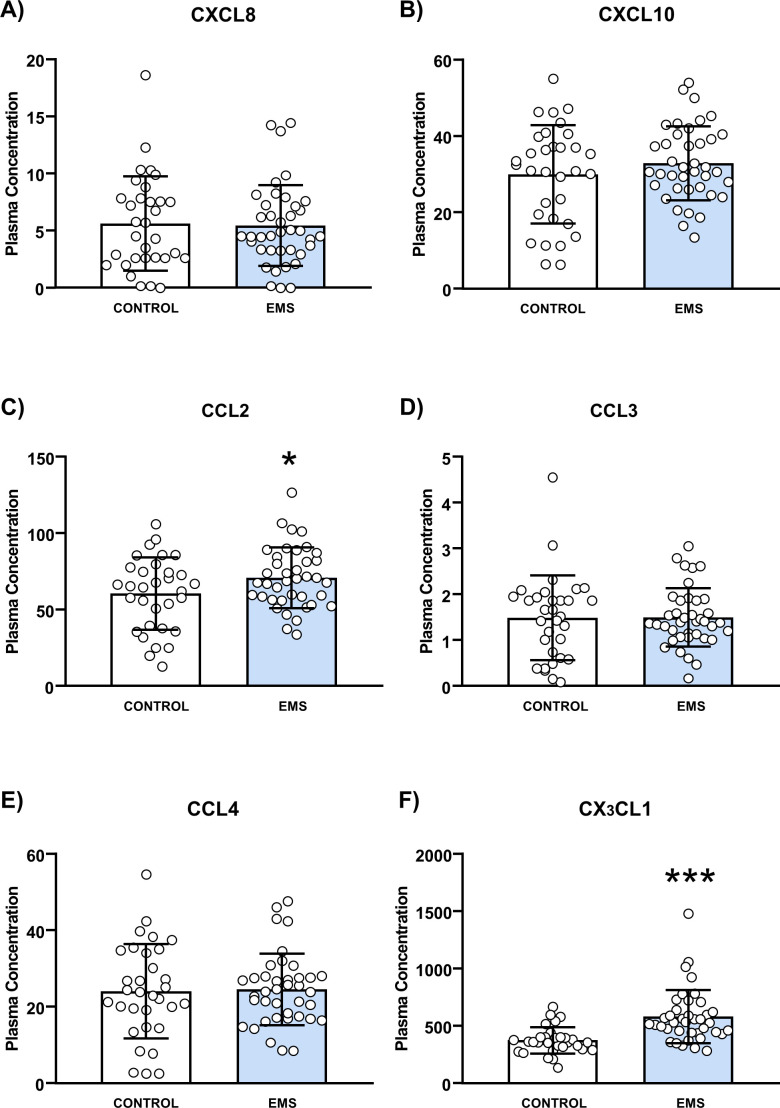
Plasma chemokine concentrations in controls and EMS personnel. **(A)** CXCL8 (pg/mL). **(B)** CXCL10 (pg/mL). **(C)** CCL2 (pg/mL). **(D)** CCL3 (pg/mL). **(E)** CCL4 (pg/mL). **(F)** CX_3_CL1(pg/mL). Concentrations are shown on the raw scale. Bars represent the mean with SD for normally distributed variables (CXCL10, CCL2, and CCL4) and the median with IQR for non-normally distributed variables (CXCL8, CCL3, and CX_3_CL1). Data were analyzed using Student’s *t* test or the Mann-Whitney *U* test according to distribution. *denotes nominal *p* < 0.05 and ***denotes nominal *p* < 0.001 compared with the control group. Numerical values and statistical results are reported in **Supplementary Table 1**.

Analysis of plasma chemokines showed significantly higher concentrations of CCL2 (*t* = 2.005, *p* = 0.049; Cohen’s *d* = 0.55, 95%CI 0.08–1.03; **Figure 2C**) and CX_3_CL1 (*U* = 219.5, *p* < 0.001; Cohen’s *d* = 1.24, 95%CI 0.72–1.75; **Figure 2F**) in the EMS group compared with the control group. After FDR correction, the between-group difference remained significant for CX_3_CL1 (adjusted *p*-value = 0.006) but not for CCL2 (**Supplementary Table 1**).

No significant group differences were detected for CXCL8 (**Figure 2A**), CXCL10 (**Figure 2B**), CCL3 (**Figure 2D**), or CCL4 (**Figure 2E**).

#### 3.2.3 Soluble adhesion molecules

**Figure 3:**
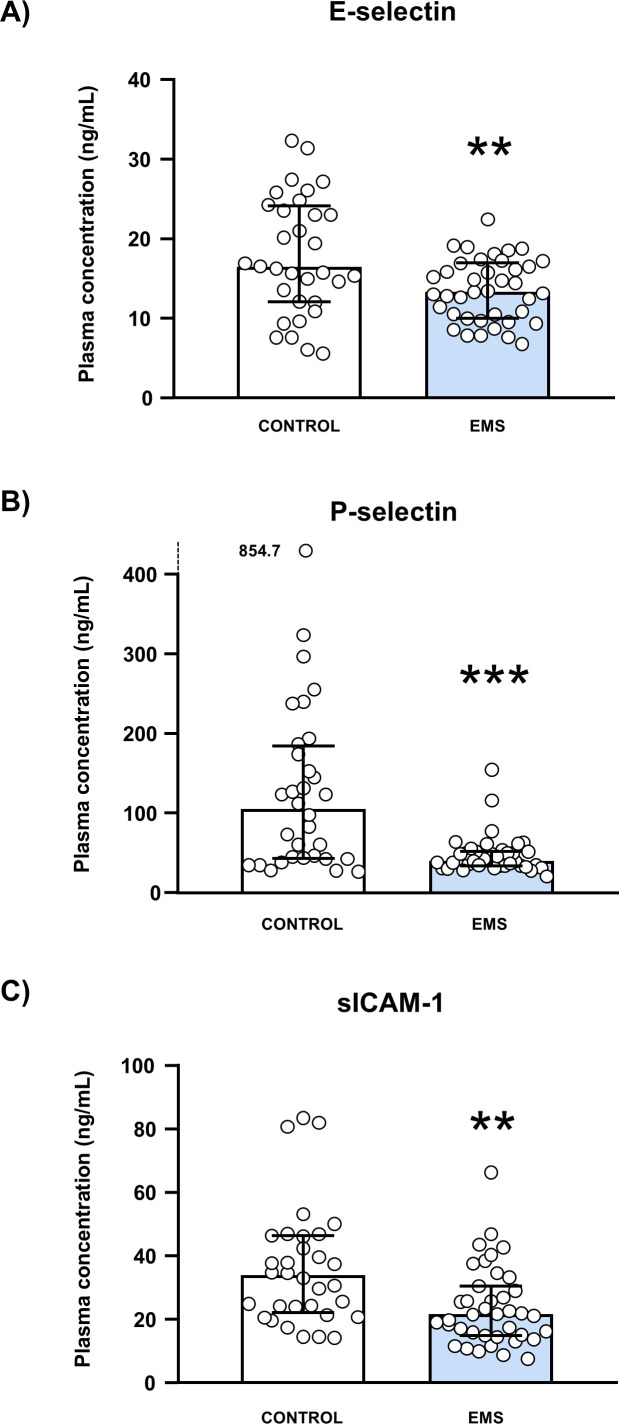
Plasma concentrations of soluble adhesion molecules in controls and EMS personnel. **(A)** E-selectin (ng/mL). **(B)** P-selectin (ng/mL). **(C)** sICAM-1 (ng/mL). Concentrations are shown on the raw scale. Bars represent the mean with SD for normally distributed variables (E-selectin) and the median with IQR for non-normally distributed variables (P-selectin and sICAM-1). Data were analyzed using Student’s *t* test or the Mann-Whitney *U* test according to distribution. **denotes nominal *p* < 0.01 and ***denotes nominal *p* < 0.001 compared with the control group. Numerical values and statistical results are reported in **Supplementary Table 1**.

In contrast to the elevated cytokine and chemokine profile, the EMS group showed lower plasma concentrations of soluble adhesion molecules. Specifically, E-selectin (*t* = 2.935, *p* = 0.005; Cohen’s *d* = -0.56, 95%CI -1.04 – -0.09; **Figure 3A**), P-selectin (*U* = 298, *p* < 0.001; Cohen’s *d* = -1.25, 95%CI -1.76 – -0.73; **Figure 3B**), and sICAM-1 (*U* = 353, *p* = 0.002; Cohen’s *d* = -0.85, 95%CI -1.34 – -0.36; **Figure 3C**) were significantly lower in the EMS group than in the control group. All three differences remained significant after FDR correction (adjusted *p*-value ≤ 0.005; **Supplementary Table 1**).

### 3.3 Correlation structure of cortisol and inflammatory mediators

**Figure 4:**
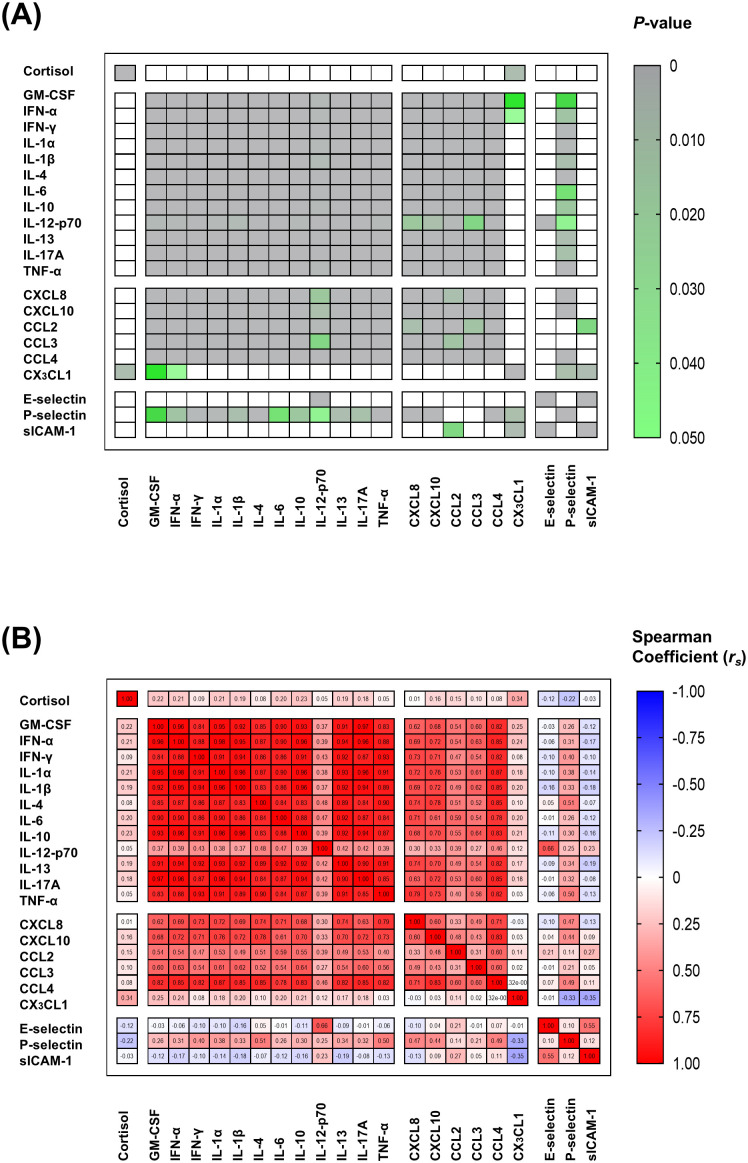
Spearman correlation analysis among cortisol and inflammatory mediators, grouped into cytokines, chemokines, and soluble adhesion molecules. **(A)** *P*-values. **(B)** Spearman coefficients. Heat maps display significant *p*-values (*p* < 0.05) and corresponding Spearman correlation coefficients (*r_s_*) ranged from –1 to 1. Correlations are exploratory, and no correction for multiple comparisons was applied.

Spearman correlation analyses were used to explore associations between cortisol and circulating inflammatory mediators and to characterize the internal structure of the inflammatory mediator network (**Figures 4A, B**). Most cytokines and chemokines showed strong positive intercorrelations, with correlation coefficients typically ranging from *r_s_* = 0.80 to 0.97 (all *p* < 0.001). This highly coordinated pattern included GM-CSF, IFN-α, IFN-γ, IL-1α, IL-1β, IL-6, IL-10, IL-13, IL-17A, TNF-α, CXCL8, CXCL10, CCL3, and CCL4.

In contrast, CX_3_CL1 showed a distinct correlation pattern. Unlike the broader cytokine–chemokine cluster, CX_3_CL1 showed weak or negligible associations with most inflammatory mediators. Similarly, E-selectin and sICAM-1 showed low correlations with most cytokines and chemokines, whereas P-selectin showed positive correlations with selected inflammatory mediators, including IL-1β, TNF-α, CXCL8, and CXCL10 (all *p* < 0.01).

Cortisol was not significantly associated with any cytokine, chemokine, or adhesion protein except for CX_3_CL1. Cortisol levels showed a positive correlation with CX_3_CL1 (*r_s_* = 0.34, *p* = 0.002), whereas correlations with the remaining inflammatory mediators were weak and non-significant. These findings suggest that cortisol was selectively associated with CX_3_CL1 rather than with generalized inflammatory activation.

We further examined these correlations separately in men and women, and observed similar inflammatory correlation patterns in both sexes (**Supplementary Figure 1**). In both groups, CX3CL1 remained weakly associated with the broader cytokine–chemokine network. However, the cortisol–CX3CL1 association was more evident among women (n = 27) than among men (n = 12), in whom it did not reach statistical significance. This finding should be interpreted cautiously given the exploratory nature of the analysis and the unequal subgroup sizes.

### 3.4 Clinical and psychological characteristics of the EMS group according to sex

Clinical, lifestyle, psychological, and physiological variables in the EMS group were analyzed according to sex (**Table 2**). In the EMS group, 28% of participants reported current daily tobacco use, and approximately 80% reported consuming more than one alcoholic drink per week. Most participants reported no current medical conditions. Seven participants were taking psychotropic medication, and nine were receiving psychological treatment.

**Table 2:** Clinical and psychological variables in the EMS group based on sex.

Variable		EMS			*p*-value
		Total	Men	Women	
Participants (n)		39	12	27	**0.001 ** ^a^
Tobacco (daily use) [n (%)]	Yes	11 (28.2)	2 (16.7)	9 (33.3)	0.286 ^a^
	No	28 (71.8)	10 (83.3)	18 (66.7)	
Alcohol [n (%)]	> 1 drink per week	31 (79.5)	8 (66.7)	23 (85.2)	0.221 ^a^
	≤ 1 drink per week	8 (20.5)	4 (33.3)	4 (14.8)	
Current medical problem [n (%)]	No	23 (59.0)	9 (75.0)	14 (51.9)	0.578 ^a^
	Trauma	7 (17.9)	0 (0.0)	7 (25.9)	
	Metabolic	5 (12.8)	2 (16.7)	3 (11.1)	
	Mental health	2 (5.1)	1 (8.3)	1 (3.7)	
	Cardiovascular	2 (5.1)	0 (0.0)	2 (7.4)	
Psychotropic medication [n (%)]	Yes	7 (17.9)	1 (8.3)	6 (22.2)	0.403 ^a^
	No	32 (82.1)	11 (91.7)	21 (77.8)	
Psychological treatment [n (%)]	Yes	9 (23.1)	1 (8.3)	4 (14.8)	>0.999 ^a^
	No	30 (76.9)	11 (91.7)	23 (85.2)	
DASS-21 [median (IQR)]	Stress	7.00 (4.00 - 10.00)	3.00 (1.00 - 7.00)	8.00 (5.00 - 10.00)	**0.010 ** ^b^
	Anxiety	4.00 (1.00 - 6.00)	1.00 (0.00 - 3.75)	5.00 (2.00 - 7.00)	**0.007 ** ^b^
	Depression	3.00 (1.00 - 5.00)	2.00 (0.00 - 3.75)	3.00 (2.00 - 6.00)	0.061 ^b^
	Total	13.25 (7.00 – 20.00)	7.00 (2.00 - 13.25)	18.00 (7.00 - 22.00)	**0.008 ** ^b^
Blood pressure (mm Hg) (mean ± SD)	Systolic	133.82 ± 18.30	144.50 ± 17.24	129.07 ± 17.00	**0.013 ** ^c^
	Diastolic	84.18 ± 10.95	90.83 ± 12.40	81.22 ± 8.98	**0.009 ** ^c^
Cortisol (nmol/L) (mean ± SD)		763.54 ± 166.03	716.72 ± 138.35	784.35 ± 175.30	0.245 ^c^

**^a^**Data were analyzed using the Fisher´s exact test or chi-squared test.

**^b^**Data were analyzed using the Man–Whitney *U* test.

**^c^**Data were analyzed using the Student’s *t* test.

IQR, interquartile range; EMS, emergency medical services; SD, standard deviation.

Bold values indicate statistical significance.

Sex-related differences were observed in psychological distress and blood pressure. Women reported higher DASS-21 stress scores (*p* = 0.010) and anxiety scores (*p* = 0.007) than men. The total DASS-21 score was also higher in women than in men (*p* = 0.008). In contrast, men showed higher systolic (*p* = 0.013) and diastolic (*p* = 0.009) blood pressure than women. No significant sex-related difference was observed in plasma cortisol levels within the EMS group.

### 3.5 Effects of groups and sex on cortisol and inflammatory mediators

To further examine group- and sex-related patterns, log_10_-transformed concentrations of cortisol and inflammatory mediators were analyzed using two-way ANOVA with group and sex as fixed factors (**Table 3**).

**Table 3:** Plasma concentrations of inflammatory mediators in the sample based on group and sex using log_10_-transformed values.

Variable	Estimated mean ^a^				Statistical analysis ^b^					
	Control		EMS		f1 (Group)		f2 (Sex)		f1 × f2	
	Men	Women	Men	Women	*F*-value	*P*-value	*F*-value	*P*-value	*F*-value	*P*-value
Log_10_ Cortisol (nmol/L)	2.81 (2.75-2.87)	2.72 (2.67-2.77) *	2.85 (2.78-2.91)	2.88 (2.84-2.93) **^&^**	13.03	**0.001**	1.00	0.321	5.31	**0.024**
Log_10_ GM-CSF (pg/mL)	2.29 (2.04-2.35)	2.01 (1.88-2.14)	2.33 (2.16-2.49)	2.30 (2.19-2.41)	8.75	**0.004**	2.33	0.131	1.20	0.278
Log_10_ IFN-α (pg/mL)	0.68 (0.53-0.84)	0.49 (0.36-0.61)	0.79 (0.63-0.95)	0.77 (0.66-0.87)	7.55	**0.008**	2.42	0.125	1.64	0.205
Log_10_ IFN-γ (pg/mL)	1.57 (1.41-1.72)	1.49 (1.36-1.62)	1.68 (1.52-1.84)	1.63 (1.52-1.74)	3.27	0.075	0.75	0.389	0.03	0.868
Log_10_ IL-1α (pg/mL)	0.62 (0.42-0.82)	0.42 (0.25-0.59)	0.75 (0.54-0.96)	0.73 (0.59-0.87)	5.84	**0.018**	1.43	0.236	0.92	0.342
Log_10_ IL-1β (pg/mL)	1.83 (1.66-2.00)	1.66 (1.52-1.80)	1.98 (1.80-2.16)	1.94 (1.82-2.06)	7.84	**0.007**	1.73	0.193	0.77	0.385
Log_10_ IL-4 (pg/mL)	1.47 (1.35-1.58)	1.39 (1.29-1.49)	1.52 (1.39-1.64)	1.51 (1.43-1.59)	2.53	0.117	0.65	0.424	0.42	0.521
Log_10_ IL-6 (pg/mL)	1.38 (1.15-1.60)	1.33 (1.15-1.52)	1.69 (1.46-1.93)	1.69 (1.53-1.84)	10.85	**0.002**	0.07	0.791	0.03	0.860
Log_10_ IL-10 (pg/mL)	0.74 (0.49-0.99)	0.52 (0.32-0.73)	0.94 (0.69-1.20)	0.92 (0.75-1.10)	6.95	**0.010**	1.07	0.305	0.83	0.366
Log_10_ IL-12-p70 (pg/mL)	2.47 (2.40-2.54)	2.40 (2.34-2.45)	2.47 (2.40-2.54)	2.42 (2.37-2.47)	0.14	0.709	3.78	0.056	0.18	0.671
Log_10_ IL-13 (pg/mL)	1.57 (1.44-1.70)	1.45 (1.34-1.55)	1.69 (1.56-1.82)	1.67 (1.58-1.76)	8.40	**0.005**	1.59	0.211	0.73	0.396
Log_10_ IL-17A (pg/mL)	1.70 (1.57-1.82)	1.51 (1.41-1.61)	1.74 (1.60-1.87)	1.70 (1.62-1.79)	4.15	**0.046**	3.73	0.058	1.84	0.180
Log_10_ TNF-α (pg/mL)	1.45 (1.33-1.58)	1.44 (1.34-1.55)	1.53 (1.40-1.66)	1.51 (1.43-1.60)	1.68	0.199	0.06	0.816	0.00	0.961
Log_10_ CXCL8 (pg/mL)	0.57 (0.28-0.86)	0.50 (0.26-0.73)	0.63 (0.31-0.94)	0.68 (0.48-0.89)	0.86	0.356	0.00	0.957	0.24	0.626
Log_10_ CXCL10 (pg/mL)	1.52 (1.41-1.62)	1.35 (1.27-1.44) *	1.47 (1.36-1.58)	1.51 (1.43-1.58) **^&^**	1.31	0.257	1.96	0.166	4.46	**0.038**
Log_10_ CCL2 (pg/mL)	1.84 (1.75-1.93)	1.67 (1.59-1.74)	1.89 (1.79-1.98)	1.81 (1.75-1.87)	5.32	**0.024**	9.75	**0.003**	1.58	0.213
Log_10_ CCL3 (pg/mL)	-0.04 (-0.21-0.13)	0.12 (-0.02-0.26)	0.22 (0.05-0.40)	0.08 (-0.03-0.20)	2.24	0.139	0.01	0.914	3.78	0.056
Log_10_ CCL4 (pg/mL)	1.42 (1.28-1.56)	1.20 (1.09-1.32)	1.40 (1.26-1.55)	1.34 (1.24-1.44)	0.85	0.360	4.96	**0.029**	1.45	0.233
Log_10_ CX_3_CL1 (pg/mL)	2.56 (2.47-2.64)	2.55 (2.48-2.62)	2.75 (2.66-2.84)	2.73 (2.67-2.79)	24.36	**<0.001**	0.13	0.724	0.03	0.858
Log_10_ E-selectin (ng/mL)	1.25 (1.16-1.34)	1.18 (1.11-1.26)	1.17 (1.07-1.27)	1.09 (1.03-1.15)	4.21	**0.044**	3.02	0.087	0.02	0.881
Log_10_ P-selectin (ng/mL)	1.93 (1.77-2.08)	2.01 (1.89-2.14)	1.65 (1.49-1.81)	1.63 (1.52-1.73)	22.56	**<0.001**	0.20	0.654	0.68	0.414
Log_10_ sICAM-1 (ng/mL)	1.62 (1.50-1.73)	1.44 (1.34-1.53)	1.38 (1.26-1.50)	1.31 (1.23-1.38)	12.74	**0.001**	6.10	**0.016**	1.05	0.310

^a^Estimated marginal mean and 95% CI of log_10_-transformed values.

^b^Data were analyzed using two-way ANOVA with group (f1) and sex (f2) as factors.

*denotes significant differences compared with control men; ^&^denotes significant differences compared with control women.

Bold values indicate statistical significance.

#### 3.5.1 Cortisol

Analysis of log_10_-transformed cortisol levels revealed a significant main effect of group (*F*_(1,74)_ = 13.03, *p* = 0.001) and a significant group × sex interaction (*F*_(1,74)_ = 5.31, *p* = 0.024). *Post hoc* comparisons indicated that cortisol levels were significantly lower in control women than in control men, whereas the EMS group showed elevated cortisol levels without significant sex-related differences.

#### 3.5.2 Cytokines and chemokines

Two-way ANOVA showed significant main effects of group on several cytokines, including GM-CSF (*F*_(1,70)_ = 8.75, *p* = 0.004), IFN-α (*F*_(1,70)_ = 7.55, *p* = 0.008), IL-1α (*F*_(1,70)_ = 5.84, *p* = 0.018), IL-1β (*F_(_*_1,70_*_)_* = 7.84, *p* = 0.007), IL-6 (*F*_(1,70)_ = 10.85, *p* = 0.002), IL-10 (*F*_(1,70)_ = 6.95, *p* = 0.010), IL-13 (*F*_(1,70)_ = 8.40, *p* = 0.005), and IL-17A (*F*_(1,70)_ = 4.15, *p* = 0.045). For all these analytes, levels were higher in the EMS group than in the control group.

Among chemokines, significant main effects of group were observed for CCL2 (*F*_(1,70)_ = 5.32, *p* = 0.024) and CX_3_CL1 (*F*_(1,70)_ = 24.36, *p* < 0.001), with higher levels in the EMS group than in the control group. A significant main effect of sex was observed for CCL2 (*F*_(1,70)_ = 9.75, *p* = 0.003), with lower levels in women than in men, and for CCL4 (*F*_(1,70)_ = 4.96, *p* = 0.029), also with lower levels in women. A significant group × sex interaction was detected for CXCL10 (*F*_(1,70)_ = 4.46, *p* = 0.038), with lower levels in control women compared with control men, whereas this sex-related difference was not observed in the EMS group.

#### 3.5.3 Soluble adhesion molecules

Two-way ANOVA also revealed significant main effects of group on E-selectin (*F*_(1,70)_ = 4.21, *p* = 0.044), P-selectin (*F*_(1,70)_ = 22.56, *p* < 0.001), and sICAM-1 (*F*_(1,70)_ = 12.74, *p* = 0.001), with lower levels in the EMS group than in the control group. A significant main effect of sex was observed for sICAM-1 (*F*_(1,70)_ = 6.10, *p* = 0.016), with lower levels in women than in men.

### 3.6 Exploratory cardiovascular-related biomarkers

As a secondary, exploratory analysis, and because the EMS group showed higher systolic blood pressure and cortisol levels, an exploratory panel of cardiovascular-related proteins was also analyzed. Overall, this panel did not show consistent group differences. Instead, most effects were sex-related, with women showing higher A2M and CRP levels than men. A significant interaction was observed for SAP, although this pattern was restricted to the EMS group. No group- or sex-related differences were detected for HPTGN or AGP.

Complete statistical results for these biomarkers are provided in the **Supplementary Results**, including Benjamini–Hochberg FDR-adjusted *p*-values for this exploratory panel (**Results S1**).

## 4 Discussion

This exploratory cross-sectional study identified an altered cytokine–chemokine and physiological pattern in EMS personnel compared with matched controls. The main findings were: (i) higher systolic blood pressure and cortisol levels in the EMS group; (ii) increased plasma concentrations of several cytokines and chemokines, including CCL2 and CX_3_CL1/fractalkine; (iii) reduced concentrations of soluble adhesion molecules; (iv) and a selective association between cortisol and CX_3_CL1, particularly among women. These findings suggest that EMS personnel may exhibit a distinct biological profile involving inflammatory and neuroendocrine–immune markers in the context of psychological distress. Among these immune findings, the higher CX_3_CL1 and the lower E-selectin, P-selectin, and sICAM-1 remained significant after correction for multiple comparisons, whereas the individual cytokine elevations and CCL2 did not and are therefore interpreted as a coherent pattern rather than robust isolated effects.

Increased plasma concentrations of several cytokines (i.e., GM-CSF, IFN-α, IL-1α, IL-1β, IL-6, IL-10, and IL-13) and chemokines (i.e., CCL2 and CX_3_CL1) suggest a broad alteration of the cytokine–chemokine profile, spanning both pro- and anti-inflammatory mediators, in the EMS group compared with controls. Extensive psychoneuroimmunology research has shown that psychological distress and related stress responses are associated with low-grade inflammation through activation of the HPA axis and downstream inflammatory pathways (31–33). Occupational studies have similarly reported elevated IL-6, IL-1β, TNF-α, and related mediators among individuals exposed to prolonged work strain, effort–reward imbalance, or interpersonal conflict (34–37). Healthcare professionals may be particularly vulnerable, with studies showing that shift work and demanding clinical environments can modulate inflammatory profiles, including IL-1α, IL-1β, IL-6, IL-10, IL-13, and CCL2 (38–40). However, findings across studies are not entirely consistent (41), suggesting that variability may be influenced by stressor type, duration, and individual resilience, occupational context, and methodological differences. Within this framework, our findings extend previous evidence by characterizing a broad cytokine–chemokine profile in EMS personnel.

In contrast to the increased concentrations of cytokines and chemokines, circulating E-selectin, P-selectin, and sICAM-1 were lower in the EMS group. Adhesion molecules participate in leukocyte recruitment, endothelial interaction, and vascular homeostasis, and changes in their circulating concentrations have been associated with endothelial activation and cardiovascular risk in clinical and metabolic populations (42). However, the interpretation of lower adhesion molecule concentrations in a relatively healthy working population is complex. This was an unexpected observation, and the interpretations proposed below remain speculative and require replication. Rather than indicating overt endothelial dysfunction, this pattern may reflect a compensatory, adaptive, or context-dependent vascular response. Psychosocial stress has been associated with impaired endothelial responsiveness and early vascular dysfunction in population studies (19), supporting the biological plausibility of a link between psychological distress, vascular regulation, and immune mediators. Nevertheless, additional longitudinal and mechanistic studies are needed to clarify whether the pattern observed here reflects adaptation, altered leukocyte–endothelial dynamics, or early vascular vulnerability.

The correlation analyses provide further insight into the organization of the immune mediator profile. Most cytokines and chemokines showed strong positive intercorrelations, forming a coordinated inflammatory cluster. These strong intercorrelations (up to *r_s_* = 0.97) are consistent with coordinated biological co-regulation, although shared analytical variance cannot be fully excluded, as most analytes were measured on the same multiplex panel. In contrast, CX_3_CL1 showed weaker associations with this broader cytokine–chemokine network and was selectively associated with cortisol. Because CX_3_CL1 was quantified in part with a separate simplex assay, its more independent pattern and selective cortisol association are unlikely to reflect shared platform variance. This finding suggests that circulating CX_3_CL1 may be regulated differently from the generalized inflammatory mediator cluster and may be more closely linked to neuroendocrine activity. Experimental studies have shown that the CX_3_CL1/CX_3_CR1 axis modulates microglial activation, neuronal stress reactivity, and behavioral adaptation under chronic stress (28, 29, 43), while clinical studies indicate that soluble CX_3_CL1 levels can change in response to acute psychological stress or targeted interventions (27, 44). Consistent with a fractalkine-linked neuroendocrine–cardiac axis, we have recently shown in mice that disruption of CX_3_CR1 signaling coordinates HPA-axis activity (ACTH and corticosterone), circulating CX_3_CL1, and cardiac injury biomarkers after adolescent stress and alcohol exposure (45). Together, these findings support the relevance of CX_3_CL1/fractalkine as a candidate chemokine marker associated with stress-related neuroimmune responses. In the present study, the selective cortisol–CX_3_CL1 association may therefore represent a peripheral marker of neuroendocrine–immune interaction rather than a generalized cortisol-driven inflammatory response.

Exploratory sex-related patterns were observed across psychological, physiological, and inflammatory measures. Women in the EMS group reported higher anxiety and stress scores, whereas men showed higher systolic and diastolic blood pressure. In addition, the association between cortisol and CX_3_CL1 appeared more evident among women. These findings may reflect sex-related differences in stress reactivity, HPA axis regulation, glucocorticoid sensitivity, and immune–endocrine communication. Previous studies have demonstrated sex-dependent differences in glucocorticoid feedback, cytokine responses, and neuroimmune signaling under psychosocial stress (46–48), and recent evidence suggests potential sex-related modulation of the fractalkine pathway (27, 49). However, these findings should be interpreted cautiously because the sex-stratified analyses were exploratory and subgroup sizes were limited and unequal.

**Figure 5:**
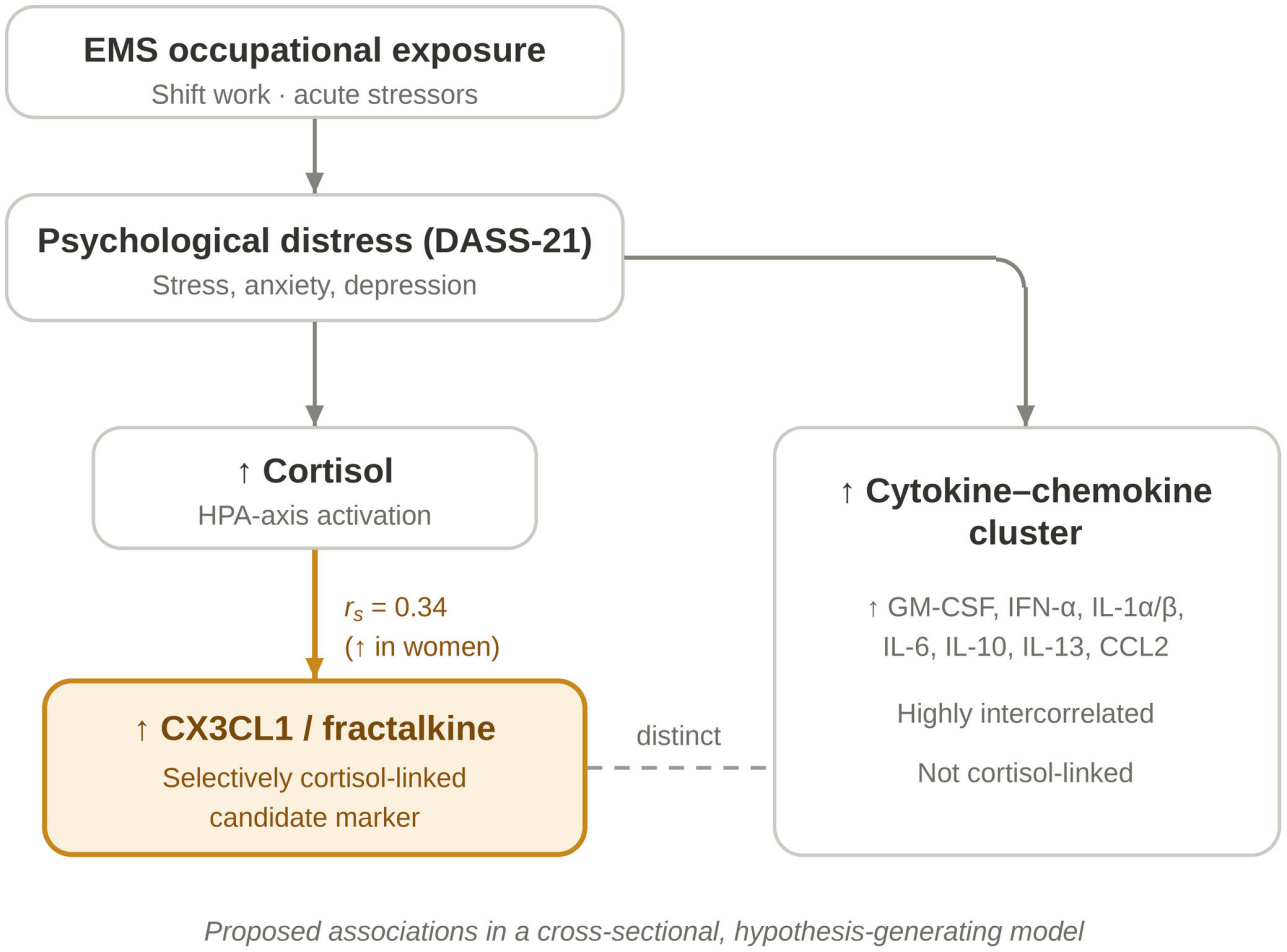
Conceptual summary of the proposed associations among EMS occupational exposure, psychological distress, cortisol, CX_3_CL1/fractalkine, and the broader cytokine–chemokine alterations. In EMS personnel, occupational exposure relates to psychological distress (DASS-21) and to HPA-axis activation with higher cortisol. Cortisol is selectively associated with CX_3_CL1/fractalkine (*r_s_* = 0.34; more evident in women), which behaves as a candidate marker distinct from the broader, highly intercorrelated cytokine–chemokine cluster (i.e., GM-CSF, IFN-α, IL-1α/β, IL-6, IL-10, IL-13, CCL2); this cluster is not associated with cortisol. The scheme depicts associations in a cross-sectional, hypothesis-generating model and does not imply causality.

From a translational perspective, the association between cortisol and CX_3_CL1 suggests that integrating endocrine and immune markers may help characterize biological responses related to psychological distress in high-demand healthcare populations. CX_3_CL1 may be particularly relevant because it exhibited a pattern distinct from the broader inflammatory cluster, supporting its potential as a candidate marker of selective neuroendocrine–immune communication. However, the present findings should not be interpreted as evidence of diagnostic or prognostic utility. Instead, they provide a hypothesis-generating basis for future studies aimed at validating whether cortisol–CX_3_CL1 relationships can help identify biological vulnerability or resilience profiles in EMS personnel (**Figure 5**).

To complement the inflammatory findings, we included an exploratory assessment of cardiovascular-related biomarkers to evaluate whether the elevated systolic blood pressure and cortisol observed in the EMS group corresponded to broader alterations in circulating cardiovascular-related proteins. Previous work has linked job strain and unfavorable working conditions with higher blood pressure and inflammatory markers such as CRP (50). In our sample, however, the cardiovascular-related biomarker panel did not show a consistent group-related pattern. Instead, most effects were sex-related, with women showing higher A2M and CRP concentrations and a sex-dependent SAP profile restricted to the EMS group, whereas no group- or sex-related differences were detected for HPTGN or AGP. Given that proteins such as A2M, CRP, SAP, and HPTGN may act as inflammatory biomarkers with prognostic value in cardiovascular disease (51), these findings suggest that cytokine–chemokine alterations may be detectable before consistent changes in more classical cardiovascular-related proteins. Longitudinal studies are needed to determine whether these immune alterations precede, accompany, or predict later cardiovascular risk in this population. Recent reviews of HPA-axis dysregulation and cortisol dynamics further link chronic stress to cardiovascular risk, providing additional biological rationale for the higher cortisol and systolic blood pressure observed here (52, 53).

### 4.1 Limitations

This study has several limitations. The cross-sectional design precludes causal inferences, and the sample size, although adequate for exploratory purposes, may limit generalizability, particularly in sex-stratified analyses. Psychological distress was assessed using the DASS-21, which captures general symptoms of stress, anxiety, and depression rather than domain-specific occupational stress. Therefore, the findings should be interpreted as reflecting psychological distress in a high-demand occupational context rather than direct effects of chronic occupational stress. The DASS-21 was administered only to EMS personnel; because the biobank controls did not complete it, no between-group comparison of psychological distress was possible, and the EMS-versus-control analysis should be read as a biological comparison. In addition, the DASS-21 reflects symptoms over the preceding week, whereas the biomarkers derive from a single fasting morning sample, so the psychological and biological measures may not capture the same exposure window.

Several lifestyle and clinical variables (e.g., smoking, alcohol use, comorbidities, and psychotropic and anti-inflammatory medication) were recorded only in EMS personnel and were unavailable for the biobank controls, so the groups could not be compared on these factors, and residual confounding cannot be excluded. Because controls were recruited from a biobank rather than from the same occupational setting, the groups may also differ in socioeconomic background, physical activity, and unmeasured background characteristics. Although blood was sampled within comparable morning windows in both groups, the exact time since awakening and sleep duration were not recorded, and other circadian factors were not fully controlled. Demographic and clinical data for the control group were obtained from biobank records and may have been measured under conditions different from those of the EMS group; accordingly, the systolic blood pressure comparison should be interpreted with caution. In addition, detailed occupational exposure data (e.g., shift type, years of service, number of night shifts, recent traumatic events, workload intensity, and burnout) were not captured and may influence the biological markers studied. Finally, the broad biomarker panel and correlation analyses increase the risk of false-positive findings. Although nominal *p* values were reported to preserve the exploratory nature of the study, the interpretation focused on coherent biomarker patterns rather than isolated associations. Therefore, weaker nominal findings, particularly those not supported by sensitivity assessment for multiple comparisons, should be interpreted as exploratory and hypothesis-generating.

Future longitudinal studies with larger and more diverse cohorts, detailed occupational and circadian assessments, and repeated biomarker measurements are needed to validate these findings and clarify the temporal relationships among psychological distress, neuroendocrine responses, and immune regulation in EMS personnel.

### 4.2 Conclusions

This cross-sectional study identifies an altered cytokine–chemokine and physiological profile in EMS personnel compared with matched controls. The EMS group exhibited increased circulating cytokines and chemokines, reduced soluble adhesion molecules, higher cortisol and systolic blood pressure, and a selective association between cortisol and CX3CL1. Unlike the broader cytokine–chemokine cluster, CX3CL1 showed a more independent profile, supporting its potential relevance as a candidate stress-sensitive chemokine linked to neuroendocrine–immune responses. Exploratory sex-related findings further suggest that psychological distress and physiological responses may differ between women and men in this high-demand healthcare population. These results should be interpreted with caution given the cross-sectional design and exploratory nature of the study, but they support future longitudinal research that integrates psychological, endocrine, and immune markers to better characterize stress-related biological risk in EMS personnel.

## Data Availability

The raw data supporting the conclusions of this article will be made available by the authors, without undue reservation.
