# Supplementary material for: Cortisol–CX3CL1 association and altered cytokine–chemokine profiles in emergency medical services personnel
Source: Front Immunol. 2026 Jul 10;17:1903713. doi: 10.3389/fimmu.2026.1903713 (PMC13395661; doi:10.3389/fimmu.2026.1903713)
Supplement: Supplementary file 1 [file DataSheet1.pdf]

Figure S1

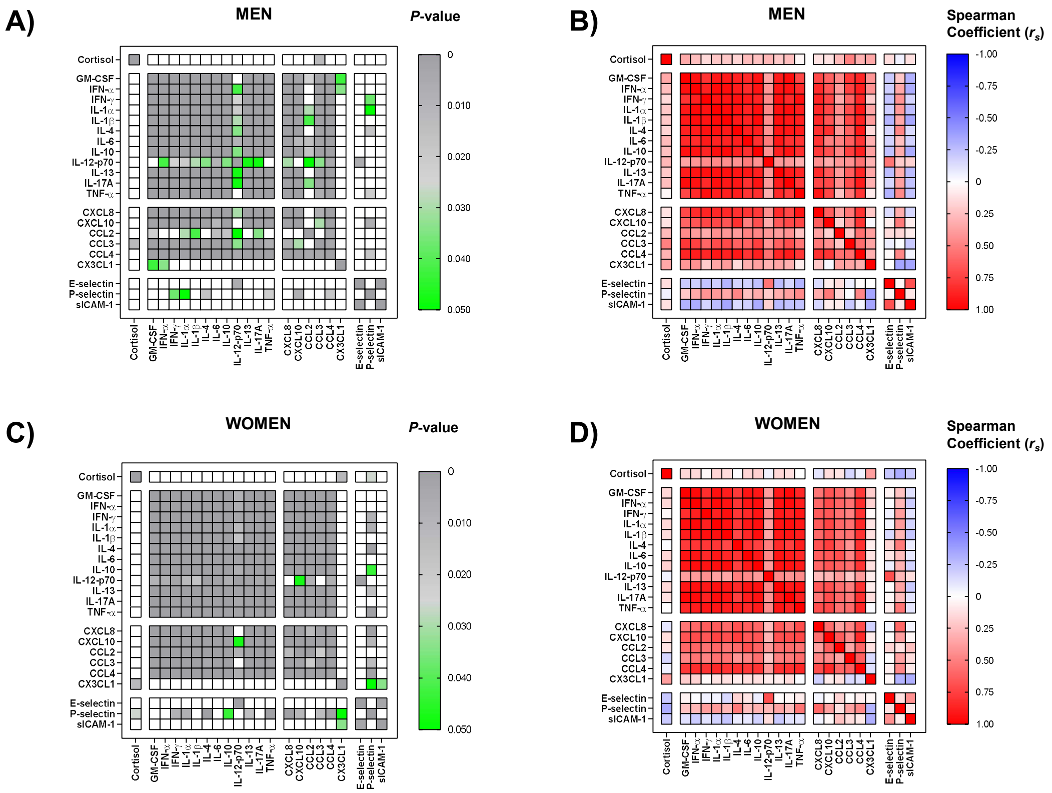

Figure S1. Spearman correlation analyses among cortisol and inflammatory mediators in men and women. (A) *P*-values for correlations in men. **(B)** Spearman coefficients for correlations in men. **(C)** *P*-values for correlations in women. **(D)** Spearman coefficients for correlations in women. Heat maps display significant *p*-values ($p < 0.05$) and corresponding Spearman correlation coefficients (r_s) ranged from –1 to 1. Correlations are exploratory, and no correction for multiple comparisons was applied.
