# Supplementary material for: Cortisol–CX3CL1 association and altered cytokine–chemokine profiles in emergency medical services personnel
Source: Front Immunol. 2026 Jul 10;17:1903713. doi: 10.3389/fimmu.2026.1903713 (PMC13395661; doi:10.3389/fimmu.2026.1903713)
Supplement: Supplementary file 2 [file DataSheet2.pdf]

Table S1. Plasma concentrations of inflammatory mediators in the sample by group.

Variable	Group		*p*-value	FDR-adjusted *p*-value
	Control	EMS		
GM-CSF (pg/mL) (mean ± SD)	166.1 ± 116.8	218.1 ± 84.25	**0.033** [a]	0.066
IFN-α (pg/mL) [median (IQR)]	4.205 (1.788-8.238)	6.060 (4.340-7.950)	**0.020** [b]	0.060
IFN-γ (pg/mL) [median (IQR)]	34.48 (16.41-65.01)	41.62 (31.30-66.99)	0.175 [b]	0.233
IL-1α (pg/mL) [median (IQR)]	3.930 (1.458-8.820)	5.200 (3.830-7.580)	**0.045** [b]	0.077
IL-1β (pg/mL) [median (IQR)]	56.6 (23.7-135.3)	83.2 (60.6-131.5)	**0.026** [b]	0.062
IL-4 (pg/mL) (mean ± SD)	31.20 ± 15.76	33.85 ± 9.96	0.412 [a]	0.449
IL-6 (pg/mL) [median (IQR)]	35.20 (12.87-68.24)	55.16 (30.91-70.81)	**0.018** [b]	0.060
IL-10 (pg/mL) [median (IQR)]	4.44 (1.79-13.30)	8.70 (5.41-16.27)	**0.020** [b]	0.060
IL-12-p70 (pg/mL) [median (IQR)]	289.4 (229.1-341.6)	272.4 (237.1-304.6)	0.471 [b]	0.471
IL-13 (pg/mL) (mean ± SD)	38.47 ± 22.13	50.19 ± 18.85	**0.018** [a]	0.060
IL-17A (pg/mL) [median (IQR)]	44.19 (25.30-67.18)	52.53 (39.58-64.81)	0.097 [b]	0.146
TNF-α (pg/mL) [median (IQR)]	32.07 (15.10-55.26)	32.95 (23.49-44.30)	0.406 [b]	0.449
CXCL8 (pg/mL) [median (IQR)]	5.105 (2.610-7.840)	4.550 (3.300-7.260)	>0.999 [b]	0.999
CXCL10 (pg/mL) (mean ± SD)	29.91 ± 12.90	32.83 ± 9.72	0.280 [a]	0.560
CCL2 (pg/mL) (mean ± SD)	60.39 ± 23.70	70.77 ± 19.93	**0.049** [a]	0.147
CCL3 (pg/mL) [median (IQR)]	1.595 (0.643-1.945)	1.390 (1.070-1.860)	0.925 [b]	0.999
CCL4 (pg/mL) (mean ± SD)	24.05 ± 12.34	24.50 ± 9.35	0.860 [a]	0.999
CX_3CL1 (pg/mL) [median (IQR)]	361.4 (296.6-403.3)	530.2 (442.0-638.3)	**<0.001** [b]	**0.006**
E-selectin (ng/mL) (mean ± SD)	17.84 ± 7.42	13.57 ± 3.93	**0.005** [a]	**0.005**
P-selectin (ng/mL) [median (IQR)]	105.5 (43.0-184.0)	39.68 (34.16-51.82)	**<0.001** [b]	**0.003**
sICAM-1 (ng/mL) [median (IQR)]	33.85 (22.09-46.40)	21.65 (14.94-30.54)	**0.002** [b]	**0.003**

[a] Data were analyzed using the Student's *t* test.
[b] Data were analyzed using the Man–Whitney *U* test.
FDR-adjusted *p*-value: Benjamini–Hochberg correction applied within each predefined analyte family (cytokines, chemokines, and soluble adhesion molecules).
Abbreviations: EMS = emergency medical services; FDR = false discovery rate; IQR = interquartile range; SD = standard deviation.
Bold values indicate statistical significance.
