# Supplementary material for: Cortisol–CX3CL1 association and altered cytokine–chemokine profiles in emergency medical services personnel
Source: Front Immunol. 2026 Jul 10;17:1903713. doi: 10.3389/fimmu.2026.1903713 (PMC13395661; doi:10.3389/fimmu.2026.1903713)
Supplement: Supplementary file 3 [file DataSheet3.pdf]

Results S1

1. Plasma concentrations of cardiovascular-related biomarkers

Variable	Control		EMS		*p*-value [a]	FDR-adjusted *p*-value
	Men	Women	Men	Women		
A2M (μg/mL) [median (IQR)]	2760 (2335-3185)	4034 (2832-5236)	2669 (1598-3739)	3124 (2382-3866)	**0.020**	**0.047**
CRP (ng/mL) [median (IQR)]	381 (76-686)	4349 (377-9075)	1451 (107-3009)	9480 (2712-16248)	**0.009**	**0.045**
SAP (ng/mL) [median (IQR)]	10474 (7906-13042)	13028 (8837-17219)	11309 (8036-14583)	8901 (5201-12601)	0.091	0.114
HPTGN (μg/mL) [median (IQR)]	1538 (908-2169)	1527 (1141-1914)	1033 (756-1310)	1481 (896-2066)	**0.028**	**0.047**
AGP (μg/mL) [median (IQR)]	2435 (2046-2824)	2621 (2051-3192)	2377 (1971-2783)	2367 (1837-2897)	0.357	0.357

[a] Data were analyzed using the Kruskal-Wallis test.
FDR-adjusted *p*-value: Benjamini–Hochberg correction applied within the cardiovascular-related protein family.
Abbreviations: EMS = emergency medical services; FDR = false discovery rate; IQR = interquartile range.
Bold values indicate statistical significance.

2. Effects of group and sex on cardiovascular-related biomarkers

Concentrations of cardiovascular-related biomarkers were $\log_{10}$-transformed and two-way ANOVA was performed with group and sex as factors.

2.1. A2M

A significant main effect of sex was observed ($F_{(1,74)} = 4.376$, $p = 0.040$), with women showing higher plasma A2M concentrations than men in both the EMS and control groups. No main effect of group or interaction was detected.

2.2. CRP

CRP levels showed a significant main effect of sex ($F_{(1,74)} = 8.018$, $p = 0.006$), with women exhibiting higher concentrations than men in both groups. No significant effects of group or interaction were found.

2.3. SAP

SAP levels showed no significant main effects of group or sex. However, there was a significant group × sex interaction ($F_{(1,74)} = 4.748$, $p = 0.033$). Post hoc comparisons indicated the following:

- Among men, SAP concentrations were significantly higher in the EMS group than in controls ($p < 0.05$).
- Among women, SAP concentrations were significantly lower in the EMS group than in controls ($p < 0.05$).
- Within the EMS group, women showed significantly lower SAP concentrations than men ($p < 0.05$).
- No sex differences were observed within the control group.

2.4. HPTGN and AGP

No significant effects of group, sex, or group × sex interaction were found for HPTGN.

Similarly, AGP concentrations showed no significant main effects or interaction.
